# Ontobee: A linked ontology data server to support ontology term dereferencing, linkage, query and integration

**DOI:** 10.1093/nar/gkw918

**Published:** 2016-10-12

**Authors:** Edison Ong, Zuoshuang Xiang, Bin Zhao, Yue Liu, Yu Lin, Jie Zheng, Chris Mungall, Mélanie Courtot, Alan Ruttenberg, Yongqun He

**Affiliations:** 1 University of Michigan Medical School, Ann Arbor, MI 48109, USA; 2 University of Pennsylvania Perelman School of Medicine, Philadelphia, PA 19104, USA; 3 Lawrence Berkeley National Laboratory, Berkeley, CA 94720, USA; 4 European Molecular Biology Laboratory-European Bioinformatics Institute, Hinxton, Cambridge, CB10 1SD, UK; 5 University at Buffalo, Buffalo, NY 14203, USA

## Abstract

Linked Data (LD) aims to achieve interconnected data by representing entities using Unified Resource Identifiers (URIs), and sharing information using Resource Description Frameworks (RDFs) and HTTP. Ontologies, which logically represent entities and relations in specific domains, are the basis of LD. Ontobee (http://www.ontobee.org/) is a linked ontology data server that stores ontology information using RDF triple store technology and supports query, visualization and linkage of ontology terms. Ontobee is also the default linked data server for publishing and browsing biomedical ontologies in the Open Biological Ontology (OBO) Foundry (http://obofoundry.org) library. Ontobee currently hosts more than 180 ontologies (including 131 OBO Foundry Library ontologies) with over four million terms. Ontobee provides a user-friendly web interface for querying and visualizing the details and hierarchy of a specific ontology term. Using the eXtensible Stylesheet Language Transformation (XSLT) technology, Ontobee is able to dereference a single ontology term URI, and then output RDF/eXtensible Markup Language (XML) for computer processing or display the HTML information on a web browser for human users. Statistics and detailed information are generated and displayed for each ontology listed in Ontobee. In addition, a SPARQL web interface is provided for custom advanced SPARQL queries of one or multiple ontologies.

## INTRODUCTION

Biomedical ontologies are sets of computer- and human-interpretable terms and relations that represent entities in the biomedical world and how they relate to each other. Ontologies are critical to biomedical data and knowledge integration, sharing and validation, as well as inferring new knowledge. To support data integration and analysis, hundreds of biomedical ontologies have been developed and are widely used. For example, since the first publication of the Gene Ontology (GO) in 2000 (1), GO has been cited by over 15 000 publications. Furthermore, many biomedical ontologies, including GO, are part of the Open Biological Ontology (OBO) Foundry, a community-based initiative which aims to establish a set of ontology development principles and incorporate ontologies following these principles in an evolving interoperable and non-redundant suite (2).

Ontologies are also the foundations of the Semantic Web and Linked Data (LD) (3) efforts. The Semantic Web is a Web of linked data defined by the standards of the World Wide Web Consortium (W3C). LD relies on Uniform Resource Identifiers (URIs) or Internationalized Resource Identifiers (IRIs) to unambiguously identify entities. When those IRIs are requested as URLs over the internet, the entities they represent can be looked up (i.e. ‘dereferenced’) using the HTTP scheme. Then the information about entities can be returned in Resource Description Frameworks (RDF) format (http://www.w3.org/TR/rdf-syntax-grammar/), such as links to other entities. This process can enhance information exchange (3). The two basic elements of LD data exchange are the definitions of individual terms and logical relations among these terms.

Ontobee is a web-based linked ontology data server that dereferences ontology terms and provides features including query, visualization and comparison of different ontologies and ontology terms. Prior to establishment of the Ontobee system in 2009, the majority of ontology term URIs pointed to the whole ontology files, or sometimes pointed to empty web pages. For example, the GO ‘biological process’ term with URI, http://purl.obolibrary.org/obo/GO_0008150, was previously pointed to the whole GO file. With the establishment of Ontobee as the default linked ontology server for GO, the term URI is now automatically linked to an Ontobee dedicated page for this term. Since 2010, Ontobee has been the default linked ontology server for most OBO library ontologies. Ontobee also includes many other resources (e.g. the Ontology of Clinical Research or OCRe) that do not belong to OBO library but are commonly used by the biomedical ontology community.

Ontobee was first presented at the 2nd International Conference on Biomedical Ontologies in 2011. Since 2011, Ontobee has been accessed by over 50 000 users. The Ontobee server has been vastly improved over the past five years. This paper systematically introduces the Ontobee system design, software development methods and various features.

## MATERIALS AND METHODS

### Ontobee three-tier architecture design and development

Ontobee is implemented using a three-tier architecture built on two HP ProLiant DL380 G6 servers that run the Redhat Linux operating system (Redhat Enterprise Linux ES 4). The three-tier client–server software architecture includes: presentation tier (i.e. user interface), logic tier (implemented by the application server) and data tier (implemented by the database sever). Users can submit ontology data queries through a web browser or an internet-based software program. The Ontobee application server is implemented using one HP server running Red Hat Linux operating system (Red Hat Enterprise Linux 5 server). The open source software program, Apache HTTP Server, is installed as the HTTP application server. PHP is used as the programming language in the web application server.

The Ontobee database server includes two types of databases: an RDF triple store database and a MySQL relational database. The RDF triple store for storing the ontologies used in Ontobee is the He Group RDF triple store developed with the Virtuoso universal server open-source system (http://virtuoso.openlinksw.com/). While ontology creators are free to choose different formats, OWL-DL is used as the default ontology format in Ontobee. The data stored in the triple store are queried using SPARQL. The He Group triple store SPARQL endpoint is publicly available for open usage (http://sparql.hegroup.org). The Ontobee MySQL database stores general metadata for each ontology (e.g. ontology developers and contact information). The data stored in the MySQL database are queried using PHP/SQL.

After a user submits an ontology-related web request or query, it is processed using PHP and SPARQL/SQL (middle-tier, application server based on Apache) against the RDF triple store and MySQL databases (back-end, database server). The query result will then be returned to the user in RDF/XML format or presented in HTML format on a web browser. Two servers are scheduled to regularly back up each other's ontology data, and the data are also backed up using an extra storage system at the University of Michigan.

### Ontology sources and data retrieval

The primary source of ontologies stored in Ontobee is the OBO Foundry Library ontology repository. Ontobee performs weekly update and automatically retrieves the most recent ontology sources, as well as their metadata from the OBO Foundry repository (http://obofoundry.github.io/). The system starts with automatic retrieval of the latest metadata available in the OBO Foundry repository, which will be used to download the newest ontology OWL sources and upload the ontology information into the Ontobee RDF triple store. Using an owl:import statement, an ontology can import other ontologies (without including the imported ontology's contents within the importing ontology file), which facilitate ontology reuse. However, the actual axioms of the thus imported resource are not merged in the importing file, which means its content is not available to Ontobee's search engine. To address this, Ontobee merges the OWL files into a single ontology. In addition, non-OBO Foundry ontologies are welcomed and can be included in Ontobee based on individual requests.

### Ontology data query and process

An Ontobee software program based on PHP and SPARQL is developed to retrieve and visualize the information about individual terms extracted from a specific ontology. The ontology hierarchical tree is assembled from the results of a set of SPARQL queries against the RDF triple store. Ontobee returns the RDF/XML formatted file for the requested term. The HTML file is embedded in an eXtensible Stylesheet Language Transformation (XSLT) (http://www.w3.org/TR/xslt) link defined in the RDF/XML file. When a user requests the term using a web browser, both the RDF/XML file and the HTML content will be retrieved but only the HTML content will be shown in the browser for easy data interpretation. The RDF/XML content is accessible via the source code of the term page. Alternatively, the RDF/XML output file can be retrieved directly by a web application program.

### Ontobee ontology term search

A keyword-based search program with auto-completion feature is available on the Ontobee website. On the client side, auto-completion is implemented using the jQuery JavaScript Library (http://api.jquery.com/) and AJAX (http://www.adaptivepath.com/ideas/ajax-new-approach-web-applications). Search over the entire set of ontologies is available on the Ontobee front page (http://www.ontobee.org). Each ontology front page and term page also has a search box that is scoped over terms from the same ontology.

### SPARQL query interface

Ontobee also includes a SPARQL query page that allows users to construct custom SPARQL queries to directly access and query ontology data stored in Ontobee through the He Group RDF triple store SPARQL endpoint (http://www.ontobee.org/sparql). A detailed tutorial with SPARQL query examples is available at http://www.ontobee.org/tutorial/sparql.

### Ontobee ontology statistics tool

The Ontobee ontology statistics tool provides statistics for all the ontologies listed in Ontobee. The tool is developed by querying the merged ontologies from the Ontobee RDF triple store database via SPARQL queries and retrieves statistics corresponding to four types of data including class, object property, datatype property and annotation property. Additionally, the Ontobee statistics tool is able to compute the original source of a term by using regular expression to recognize the prefix used in its URI.

### Ontology download

The OWL source file of the ontology can be viewed and downloaded by following the ontology Download link on the cover webpage of an ontology. A list of all classes and properties for each ontology can be downloaded from the Ontobee homepage in Excel or tab-delimited format.

### Software download and license

The source code of Ontobee is available under an Apache License 2.0 license and can be downloaded from the Ontobee GitHub repository (https://github.com/OntoZoo/ontobee). The University of Michigan is the license holder of the Ontobee software.

## RESULTS

### Ontobee system

The Ontobee system design has continuously evolved since its initial release in 2008, when it was first used to visualize the Vaccine Ontology (VO) in 2008 (4). After its success, Ontobee was updated to provide linked data for the Ontology for Biomedical Investigation (OBI) (5). Ontobee development was benefited from many encouragements, suggestions and discussions of the OBO Foundry ontology development community. By the end of 2010, Ontobee was chosen to be the default Linked Data Server for most OBO Foundry Library ontologies. Over the past few years, Ontobee has maintained and improved several functions including ontology term browsing and dereferencing to obtain related information, intuitive term searching from one ontology or across various ontologies and SPARQL queries for advanced data retrieval and analysis.

The OBO Foundry Technical Working Group generated an OBO PURL ontology term IRI dereferencing policy (http://obofoundry.org/docs/OBOPURLDomain.html). Its implementation results in direct linkage of the OBO library ontology terms to the Ontobee server.

**Figure 1.**
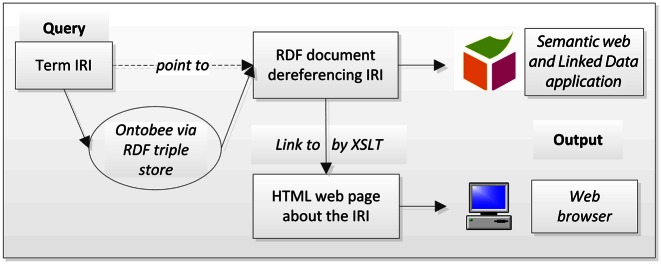
Basic Ontobee system design. An ontology term Internationalized Resource Identifier (IRI) query will be processed by Ontobee system against the Resource Description Frameworks (RDF) triple store, and then Ontobee will return an RDF document dereferencing the term IRI. This RDF document can be directly output for a Semantic web and Linked Data application. If the ontology term IRI query is requested by a web browser, the eXtensible Stylesheet Language Transformation (XSLT) statement in the RDF document will render an HTML page to display the information of the term IRI. See more explanation in the text.

The development of the Ontobee ontology term IRI dereferencing strategy was inspired by the prototype developed by the OBI developers in 2008 (6). The final version of the Ontobee ontology term IRI dereferencing system is illustrated in Figure 1. Basically, with a single ontology term IRI query, Ontobee outputs different results depending on different request methods (Figure 1). Specifically, a query for an ontology IRI term from a web browser or an http user for a Semantic Web application will send a request to the Ontobee server. Once a request is received, the Ontobee server will issue a SPARQL query against a RDF triple store and return an RDF document that dereferences the IRI. The RDF document will be returned to the Semantic Web & LOD application (no web browser involvement). For a web browser, the browser notices the XSL stylesheet and returns the HTML with an XSLT wrapper. The web browser applies that to the RDF, gets the HTML and renders the HTML to the user (Figure 1). As a result of this strategy, Ontobee is able to combine an RDF output for web applications with a HTML output for web browsing and searching.

### Ontology statistics in Ontobee

As of July 31, 2016, Ontobee provides access to more than 180 ontologies with over 4 million ontology terms. Table 1 lists representative ontologies stored in Ontobee. Detailed statistics about Ontobee resources are available at: http://www.ontobee.org/ontostat. Overall, Ontobee stores 131 OBO Foundry Library ontologies (2). The ontologies available in Ontobee cover different domains in the areas of biology (e.g. anatomy, gene, protein), biomedical sciences (e.g. biomedical investigations, reagents) and health (e.g. clinical measurements, clinical statistics).

**Table 1.** Summary of selected ontologies covered in Ontobee

Ontology Names	Number of terms
***OBO Foundry ontologies***	
ChEBI (Chemical Entities of Biological Interest)	102 458
DOID (Human Disease Ontology)	9293
GO (Gene Ontology)	46 814
OBI (Ontology for Biomedical Investigation)	3386
PATO (Phenotype Ontology)	2571
PO (Plant Ontology)	1786
PR (Protein Ontology)	296 887
XAO (Xenopus anatomy and development)	1560
ZFA (Zebrafish anatomy and development)	3113
***Selected OBO Library ontologies***	
CL (Cell Type Ontology)	6642
CLO (Cell Line Ontology)	40 987
IDO (Infectious Disease Ontology)	639
IAO (Information Artifact Ontology)	326
NCBITaxon (NCBI organismal classification)	1 410 486
OAE (Ontology of Adverse Events)	5739
VO (Vaccine Ontology)	4805
***Selected non-OBO ontologies***	
NDF-RT (National Drug File Reference Terminology)	76 068
OCRe (Ontology of Clinical Research)	676

### Ontology term query and result visualization in Ontobee

**Figure 2.**
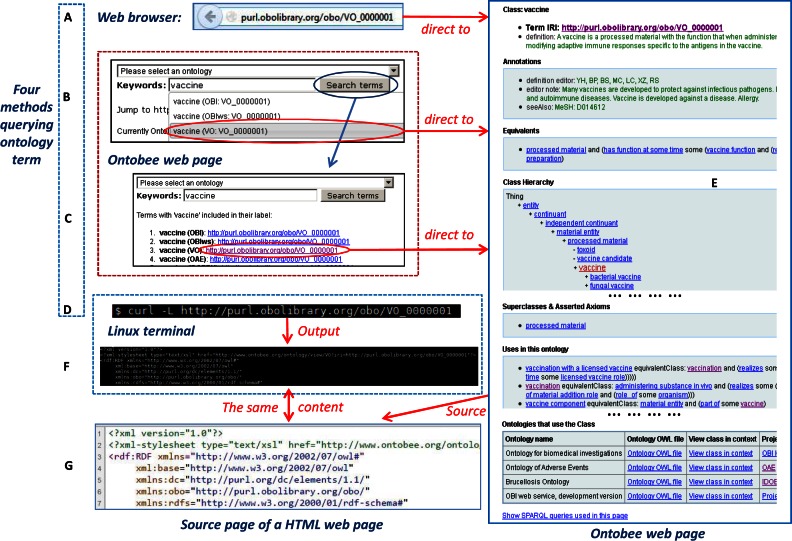
(**A–D**) Ontobee dereferencing of VO term ‘vaccine’ (VO_0000001). Different approaches can be used for dereferencing ontology URIs in Ontobee. Using a web browser, a direct browser query of an (**A**) ontology URI or an (**B** and **C**) Ontobee website query could lead to the HTML visualization page of the (**E**) ontology URI. (**B**) The Ontobee query builder allows a keyword-based simple textual search. As the user types, commencing on the third character, (**B**) a drop-down menu with terms whose label (or synonym) contains the string typed so far will show up. (**E**) A selection of a specific term in the drop-down menu will then lead to the web page dereferencing the ontology term. Alternatively the ‘Search terms’ tab can be clicked to obtain a page that lists all matches, sorted in order to first show terms that start with the search string, (**C**) shortest to longest. (**E**) A selection of a term on this page also leads to page. The HTML web page of the (**E**) term URI shows different levels of details about the ontology term. (**D**) If a non-browser web program (like a Linux terminal command call using the ‘curl’ command) is used, or if the source of the web visualization page is checked, the content of the deferenced URI is provided with the (**F** and **G**) RDF/OWL format. The contents of (**F**) in the Linux terminal and (**G**) in a web browser are the same.

Figure 2 demonstrates how Ontobee supports the query and display of individual ontology terms. There are multiple query methods available in Ontobee, including: (i) a direct Web browser query using an ontology IRI (Figure 2A), (ii) a simple keyword search on an Ontobee webpage and selection of a popup item (Figure 2B) and (iii) a simple keyword search and selection from a list of search results on a new Ontobee Web page (Figure 2C). Alternatively, Ontobee supports programmatic access. For example, in a Linux terminal, a ‘*curl*’ command can be used to directly query an ontology term URL (Figure 2D).

In terms of query outcomes, the results shown on a Web browser (Figure 2E and F) or in a Linux/Unix terminal are different (Figure 2G). To illustrate the underlying mechanism, we can start with the explanation of the OBO Foundry PURL domain policy (http://obofoundry.org/docs/OBOPURLDomain.html). The OBO policy uses the ontology term IRI format of http://purl.obolibrary.org/obo/DOMAIN_xxxxxxx. For example, the VO term ‘vaccine’ has the IRI of http://purl.obolibrary.org/obo/VO_0000001. When this term IRI is requested over the internet, it is directed to the Ontobee page: http://www.ontobee.org/ontology/VO?iri=http://purl.obolibrary.org/obo/VO_0000001. The link used in this Ontobee implementation example is constructed to specify the abbreviation of a particular ontology (e.g. VO) and the IRI of a specific term (e.g. http://purl.obolibrary.org/obo/VO_0000001) in this ontology. The direct output of this Ontobee page is an RDF/XML file as shown in the Figure 2F and G. The RDF/XML file provides related information about the URI as extracted from the Ontobee RDF triple store. The second line of the RDF/XML file contains a tag as follows:

<?xml-stylesheet type="text/xsl" href="http://www.ontobee.org/ontology/view/VO?iri=http://purl.obolibrary.org/obo/VO_0000001"?>

This line specifies an XLST stylesheet in which a HTML reference is embedded. When the query is performed in a Web browser, the HTML reference embedded in this XLST stylesheet functions and displays the HTML version of dereferenced ontology term information (Figure 2E). The HTML web page contains a user-friendly display of the term information. Various types of information are provided, including basic term IRI, term definition, various term annotation information, equivalent class definition, class hierarchy, superclasses and asserted axioms, usages, disjoints and a link ‘Show SPARQL queries used in this page’ (Figure 2E).

### Ontobee statistics program

The Ontobee statistics program (http://www.ontobee.org/ontostat) extracts statistical information of one or more ontologies from Ontobee and displays the results using a simple but comprehensive table-format in the website. The Ontobee statistics program cover page provides the statistics of all the ontologies listed in Ontobee. Once you click on a specific ontology link from the Ontobee statistics page, you can obtain detailed information about its statistics. In addition, the same statistics page for an ontology can be accessed directly by clicking ‘Detailed Statistics’ on the cover page of the specific ontology in Ontobee.

### Ontobee SPARQL query and RDF triple store usage

The Ontobee SPARQL query program (http://www.ontobee.org/sparql) is one popular program for those who have SPARQL query experience and would like to retrieve more complicated ontology information with advanced SPARQL query scripts.

The Ontobee RDF triple store has been used in OntoFox, a web server that supports ontology reuse by allowing users to input terms, fetch selected properties, annotations and certain classes of related terms from source ontologies, and save the results using the RDF/XML serialization of the OWL (7). The Ontobee RDF triple store has also been used for development of Ontodog, a web application that generates ontology views (8).

## DISCUSSION

Ontobee is popular biological and biomedical ontology browser. In addition to ontology browsing, many ontology developers and users also use Ontobee to search for ontology terms and check ontology term usages. The Ontobee SPARQL program is also used to make advanced ontology queries and analysis. As the default linked data server for most OBO Foundry Library ontologies since 2010, Ontobee has contributed significantly to the widely accepted OBO Foundry initiative.

To the best of our knowledge, Ontobee is the first software program that implements the XSLT/SPARQL-based ontology term URI dereferencing strategy (Figure 1). Different from the conventional content negotiation method (https://www.w3.org/TR/cooluris/#conneg), the XSLT-based strategy was first proposed in 2008 by OBI developers (6). The central idea of this design pattern is to dereference ontology term URIs with a default RDF output, which includes an HTML web link embedded in an XSLT statement, and when a Web browser is accessed, an HTML output for web visualization would be provided. Ontobee is the first, and so far the only, web program that implements such a strategy. One benefit of this strategy is that it does not require the users to remember and enter two different HTTP links. Although different methods may be used to implement the XSLT prototype proposed in the 2008 paper (6), Ontobee initiated the usage of a RDF triple store system and its related methods so that dynamic generation of millions of different pages becomes possible.

The most commonly used ontology browsers other than Ontobee in biomedical fields include the Ontology Lookup Service (OLS) (9), AberOWL (10) and the National Center for Biomedical Ontology (NCBO) BioPortal browser (11). Ontobee is developed using PHP and SPARQL. OLS, BioPortal and AberOWL are developed using JAVA. OLS provides lookup service and browsing capability for the OBO Format ontologies in the OBO Library (2). AberOWL provides a framework for automatically accessing ontology information (http://aber-owl.net) (10). NCBO BioPortal is the largest biomedical ontology repository and ontology browser system. Like Ontobee, these three ontology browsers also support user query, ontology hierarchical tree display and ontology term content visualization.

In terms of generating RDF output that dereferences an ontology term URI, OLS and AberOWL do not provide such service. The NCBO developers utilizes NCBO Resource Index for ontology term URI dereferencing (12). Both Bioportal and NCBO, use Java technology and a Representational State Transfer (REST) web service interface. The NCBO approach separates the ontology web browser and RDF output using NCBO BioPortal ontology web browser and Resource Index, respectively (11). In contrast, Ontobee combines these two features together in a lightweight system.
